# Elicitation enhances the production of friedelin and epifriedelanol in hairy root cultures of *Cannabis sativa* L.

**DOI:** 10.3389/fpls.2023.1242584

**Published:** 2023-08-11

**Authors:** Khwanlada Kobtrakul, Dolly Rani, Asma Binalee, Pattarapol Udomlarp, Tatiya Srichai, Wanchai De-Eknamkul, Sornkanok Vimolmangkang

**Affiliations:** ^1^ Pharmaceutical Sciences and Technology Program, Faculty of Pharmaceutical Sciences, Chulalongkorn University, Bangkok, Thailand; ^2^ Department of Pharmacognosy and Pharmaceutical Botany, Faculty of Pharmaceutical Sciences, Chulalongkorn University, Bangkok, Thailand; ^3^ Herbal and Phytochemical Testing Laboratory Center (HPTLC), Chulalongkorn University, Bangkok, Thailand; ^4^ Research Cluster for Cannabis and its Natural Substances, Chulalongkorn University, Bangkok, Thailand; ^5^ Center of Excellence in Plant-Produced Pharmaceuticals, Faculty of Pharmaceutical Sciences, Chulalongkorn University, Bangkok, Thailand

**Keywords:** hemp, marijuana, kanja, hairy root, triterpenoid, plant tissue culture, biotic stress, anti-inflammation

## Abstract

*Cannabis sativa* L. (hemp) has a global distribution and social impact, and it is widely used as a medicinal plant, food ingredient, and textile fiber. Its roots have received less attention than other parts, especially the inflorescence, leaves, and shoots. Triterpenoids, including friedelin and epifriedelanol, have been found in hemp roots, and their anti-inflammatory effects have been reported. In this study, the potential enhancement of triterpenoid accumulation in the roots of *C. sativa* by elicitation was examined. Hairy roots were successfully established, and they contained 2.02-fold higher triterpenoid levels than natural roots. Furthermore, hairy roots treated with 75 μM salicylic acid had 1.95-fold higher friedelin levels (0.963 mg/g DW) and 1.4-fold higher epifriedelanol levels (0.685 mg/g DW) than untreated hairy roots. These results suggested that the elucidation of hairy root cultures using an optimized elicitor could represent an alternative strategy to produce the valuable triterpenoids friedelin and epifriedelanol.

## 1 Introduction

Hemp (*Cannabis sativa* L.) belongs to the Cannabaceae family. It has been used as a food additive, animal feed additive, textile fiber, and folk medicine ingredient since ancient times (Fiorini et al., 2019), and it is widely cultivated globally. The phytochemical components of hemp include carbohydrates, fatty acids, stilbenoids, flavonoids, phenols, alkaloids, terpenoids, and cannabinoids (Brighenti et al., 2017), many of which are abundantly produced in glandular trichomes by female hemp inflorescences (De Backer et al., 2009). Hemp is currently popular because all of its parts can be used for multi-purpose applications. In general, the inflorescence, leaves, stems, and seeds of hemp are most widely used, whereas the roots have received less attention.

**Figure 1:**
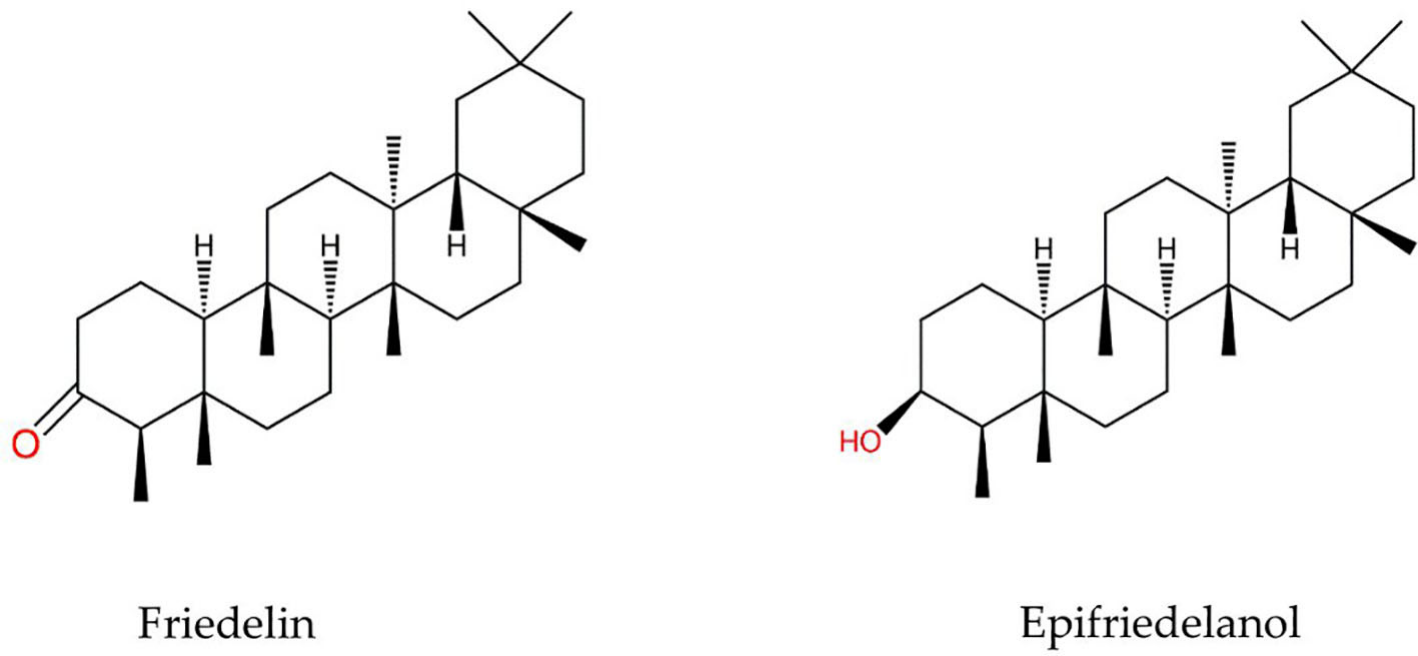
Structures of friedelin and epifriedelanol.

As such, little information about the constituents and biological activities of hemp roots is available in the literature. Hemp roots have been historically used to treat inflammation, fever, gout, arthritis, joint pain, and skin burn (Ryz et al., 2017). Furthermore, the aqueous extract of the roots of *C. sativa* exhibited anti-inflammatory and anti-asthmatic activity in mice (Lima et al., 2021; Menezes et al., 2021). In addition, the ethanolic extract of aeroponic cannabis roots displayed anti-oxidant and anti-inflammatory activity (Ferrini et al., 2022). The roots contain distinct active compounds from other parts of the plant, such as the triterpenoids friedelin and epifriedelanol (**Figure 1**), which were first characterized in 1971 (Slatkin et al., 1971). Friedelin is the most prominent triterpenoid in hemp roots, and it possess anti-diarrheal, (Antonisamy et al., 2015b) anti-inflammatory, analgesic, antipyretic (Antonisamy et al., 2011), anti-ulcer (Antonisamy et al., 2015a), and anti-microbial effects (Kuete et al., 2007a; Kuete et al., 2007b). Meanwhile, epifriedelanol was detected in smaller amounts in *C. sativa*, and its anti-oxidant and anti-inflammatory activity has been reported (Ferrini et al., 2022).

The biosynthesis of active compounds in naturally grown plants is directly affected by species and environmental factors. Furthermore, plants can be easily contaminated by heavy metals and pesticides from the soil. Therefore, plant tissue culture technologies have been established to overcome these issues. Currently, hairy root culture is a potential alternative method for the production of various classes of secondary metabolites. Hairy roots usually grow faster and display greater potential to produce valuable metabolites than wild-type roots (Giri and Narasu, 2000), and they can also be elicited with both biotic and abiotic agents to increase the production of bioactive metabolites (Park et al., 2020; Mahendran et al., 2021). The main biotic elicitors used for secondary metabolite production are methyl jasmonate (MeJA), jasmonic acid, acetylsalicylic acid, chitosan (CHI), coronatine, pectin, salicylic acid (SA), and yeast extract (YE) (Alcalde et al., 2022). Furthermore, these agents can increase terpene accumulation in hairy roots (Liang et al., 2012; Sun et al., 2012; Kim et al., 2013; Sharmila and Subburathinam, 2013; Li et al., 2016; Qiu et al., 2021). There are some reports on the development of hairy root induction in *C. sativa* and the determination of choline, atropine and cannabinoids production in these roots (Wahby et al., 2006; Wahby et al., 2013; Farag and Kayser, 2015; Berahmand et al., 2016). However, there is no report on triterpenoid production in hairy root cultures of *C. sativa*. Furthermore, the growth of wild-type *C. sativa* requires at least 3 months, and it is difficult to separate its roots from the soil. Therefore, to quickly obtain high levels of triterpenoids, this study investigated the ability of four elicitors, namely SA, MeJA, CHI, and YE, to enhance friedelin and epifriedelanol production in the hairy roots of *C. sativa* as a potential alternative approach.

## 2 Materials and methods

### 2.1 Plant material

The *C. sativa* variety Siam CA was obtained in July, 2021 from the Center of Plant-produced Biopharmaceutical for Dentistry, Faculty of Dentistry, Chulalongkorn University (Bangkok, Thailand) in collaboration with Leapdelab Co. Ltd. (Samut Prakan, Thailand). It was deposited at the Department of Botany, Faculty of Science, Chulalongkorn University (Bangkok, Thailand) under H.B. no. 17260 (BCU).

### 2.2 Chemicals and reagents

Gamborg B-5 basal medium, Murashige and Skoog (MS) medium (Murashige and Skoog, 1962), yeast extract beef broth (YEB), Agargellan™, and cefotaxime were obtained from PhytoTechnology Laboratories (Lenexa, KS, USA). YE powder was obtained from HiMedia (Maharashtra, India). MeJA (1 g/mL in water), CHI (from shrimp shells), vanillin, and acetosyringone were purchased from Sigma-Aldrich (St. Louis, MO, USA). SA was procured from Ajax Fine Chem (Sydney, Australia). The friedelin analytical standard was obtained from Extrasynthese (Genay, France), and the epifriedelanol analytical standard was acquired from Biopurify (Sichuan, China). Glacial acetic acid, sulfuric acid, toluene, dichloromethane, and ethyl acetate were obtained from Merck (Darmstadt, Germany). Chloroform was procured from Labscan (Bangkok, Thailand). For DNA isolation and amplification, a DNAsecure Plant Kit (Tiangen, Beijing, China), Taq Phusion polymerase, and GeneRuler 1 kb Plus DNA Ladder (Thermo Fisher Scientific, Waltham, MA, USA) were used, and the primers were synthesized by Macrogen (Seoul, Korea).

### 2.3 Hairy root induction and growth curve analysis

The ATCC 43057 strain of *Rhizobium rhizogenes* (ATCC Manassas, USA) was cultured in YEB containing rifampicin (50 mg/L) and shaken at 180 rpm at 28°C for 24 h. After reaching OD_600_ of 0.6, the bacterial suspension was then centrifuged at 5000 rpm for 15 min. The bacterial pellet was resuspended in liquid B5 medium containing 100 µM acetosyringone (OD_600_ of approximately 0.4).

**Figure 2:**
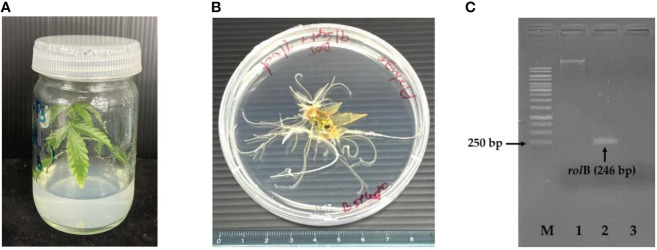
Seedling of *C.sativa* at 14 days **(A)**, initial hairy roots of *C. sativa* at 16 days **(B)** and PCR analysis of *rolB* from hairy roots **(C)**. M, GeneRuler 1kb Plus DNA Ladder; 1, genomic DNA; 2, transgenic hairy roots; 3, negative control.

*In vitro* germinated *C. sativa* seedlings (**Figure 2A**) were used as explants for hairy root induction. Aseptic seeds were inoculated in MS medium (Murashige and Skoog, 1962) supplemented with 0.5% Agargellan and 3% sucrose. The fully germinated seedlings (14 days old) with expanded leaflets were further used. Petiole explants with three leaflets were submerged in the *R. rhizogenes* culture and stirred for 15 min. The excess suspension of *R. rhizogenes* was then removed using sterile paper. Infected explants were co-cultured in B5 medium in the dark for 2–4 days. To remove the residue of *R. rhizogenes*, they were transferred to fresh B5 medium supplemented with 500 mg/L of cefotaxime for one week, then sub-cultured to the new medium with gradual reduction of cefotaxime concentration each week at 250 and 100 mg/L until no sign of bacterial re-growth observed. The explants were incubated on cefotaxime-free and auxin-free B5 medium in darkness until the initial hairy roots appeared. The induced hairy roots (approximately 2–5 cm) from single explants were cut and kept as single clones. The hairy roots were incubated on the solid medium in the dark at 25°C and sub-cultured every 14 days for four generations, and they were transferred into the liquid medium in the dark at 25°C and shaken at 150 rpm. Subcultures were performed for five generations before use in the elicitation experiment. To analyze the growth curve, fresh hairy roots were harvested and washed with distilled water, and the water was removed by suction filtration. The fresh weight was recorded on days 0, 4, 8, 12, 16, and 20 after inoculation from three independent experiments.

### 2.4 The presence of the rolB gene in hairy roots

Genomic DNA was extracted from hairy roots using the DNASecure Plant Kit according to the manufacturer’s instructions. The presence of *rolB*, which is a specific gene in transgenic roots, was confirmed in hairy roots by PCR. PCR was performed using Phusion Taq polymerase following the manufacturer’s protocol. The 246-bp fragment of *rolB* was amplified using specific primers (forward primer, 5′-GAT-ATC-CCG-AGG-GCA-TTTT-T-3′; reverse primer, 5′-GAA-TGC-TTC-ATC-GCC-ATT-TT-3′). Reaction mixtures (50 μL) were constructed in the thermal cycler (T100™, Bio-Rad, Hercules, CA, USA) using 100 ng of hairy root genomic DNA as a template. PCR was performed as follows: initial denaturation 95°C for 5 min; 30 cycles of denaturation at 95°C for 30 s, annealing at 58°C for 30 s, and extension at 72°C for 30 s; and final extension at 72°C for 5 min. The genomic DNA of native roots was used as a negative control. Finally, the PCR products were checked on 1% agarose gel and observed using a gel documentation system (Model no: Universal Hood II, Bio-Rad, USA).

### 2.5 Elicitor treatments

The stock solution of SA was prepared using 20% ethanol. The stock solution of CHI (minimum 85% deacetylated) was prepared in glacial acetic acid by gentle heating at 60°C, and the solution was brought to the 10 mL as a final volume with ultrapure water. The control for CHI elicitation was 1% acetic acid. YE was dissolved in water. The MeJA solution was initially diluted in methanol and then introduced into the hairy root cultures. The MeJA, SA, and YE solutions were filtered (0.22 µm) before addition to the medium, whereas the CHI solution was autoclaved before use. Each elicitor was applied to the root culture 10 days after subculture, representing the linear growth curve and leading to the best production of bioactive compounds of hairy root cultures. The MeJA, SA, and YE stock solutions were introduced to the culture medium to attain final concentrations of 25, 50, 75, and 100 µM. The CHI stock solution was prepared at 100 mg/mL and used at concentrations of 25, 50, 75, and 100 mg/L. Then, the culture was shaken at 150 rpm in the darkness for another 3 or 6 days (Mahendran et al., 2021). In addition, the elicitors’ solvents were tested on the root culture to ensure non-interference of the solvents on the elicitation. To harvest the hairy roots, the culture medium was filtered and kept to check the presence of the target metabolites before discard. All hairy root samples were dried in an oven at 50°C overnight and used for qualitative and quantitative analyses. The experiment was performed with three replicates.

### 2.6 Triterpenoid extraction for chemical analysis

The native roots of *C. sativa* variety Siam CA were collected and washed thoroughly after harvesting its inflorescences. The hairy roots were collected separately from the spent medium by filtering through a Whatman™ filter paper. The solution was lyophilized to dry powder. The roots were dried in the oven at 50°C. Both native root and hairy root samples were ground. Then, 0.1 g of each dried powder sample and the lyophilized powder of the medium were extracted with 1 mL of chloroform, sonicated for 30 min, and centrifuged to obtain the supernatant. The residue of the powder was then extracted again using the aforementioned extraction procedure. Finally, the supernatants were combined, centrifuged, and evaporated to obtain the crude extract.

To prepare a test solution for high-performance thin layer chromatography (HPTLC), the crude extract was resuspended in 50 μL of methanol:chloroform (9:1). For gas chromatography flame ionization detector (GC-FID), the crude extract was resuspended in 200 μL of chloroform.

### 2.7 Chemical profile and triterpenoid screening using HPTLC

Triterpenoid profiling was investigated using HPTLC. An applicator (Linomat 5, CAMAG, Muttenz, Switzerland) was used to apply 5 μL of the test solutions and 4 μL of analytical standards (1 mg/mL friedelin and epifriedelanol) onto silica gel 60 F254 HPTLC glass plates (10 × 20 cm^2^; Merck). All samples were applied to plates as 8-mm bands separated by 11.4 mm and positioned 8 mm from the lower edge and 20 mm from the left plate edge. The mobile phase was a mixture of toluene, dichloromethane, and ethyl acetate (8:1:0.5 v/v/v), and it was developed using a CAMAG automatic development chamber 2. All images were recorded using the CAMAG TLC Visualizer 2. Plates were subjected to post-chromatographic derivatization using 1% vanillin reagent in ethanol. The plates were heated on a hot plate at 105°C, and then images were captured after derivatization. The analysis of all results was controlled by VisionCATS software (Version 3.0.20196.1, CAMAG)

### 2.8 Quantification of friedelin and epifriedelanol

The content of both triterpenoids (friedelin and epifriedelanol) was analyzed by GC-FID using an Agilent HP-5MS GC column (5 HP-5 30 m × 320 μm × 0.25 μm; Agilent Technologies, Santa Clara, CA, USA). Helium was used as the carrier gas with a flow of 1.5 mL/min. A sample volume of 1.0 μL was injected with the injector and FID temperatures set at 280 and 320°C, respectively. The analysis temperature program was as follows: 0–5 min at 150–280°C with a rate at 50°C/min, maintained at 280°C for 5–25 min, and then decreased to 150°Cover 5 min at a rate at 50°C/min.

The friedelin and epifriedelanol standards were dissolved in 100% chloroform at a stock concentration of 1 mg/mL. The working standard solutions were diluted to generate five concentrations in the range of 10–200 μg/mL. The amount of both standards was quantified from a calibration curve prepared by linear regression analysis in triplicates. The linear regression equation (y = mx + b) was examined by plotting the peak area (y) and quantity (x) of each standard. The correlation coefficients of the linearity exceeded 0.996. The validation method was performed to ensure the reliability of the method. The accuracy was investigated by recovery studies. The HR was extracted with chloroform three times, and its powder was used as the matrix in the recovery studies. The recovery studies were performed by standard addition method. Each standard reference at low, medium, and high level was added to the matrix extract and were again analyzed. The % recovery was accepted following the ICH guideline, and the average percentage recovery should be in a range of 95–105% (European Medicines Agency, 2006). Also, the limit of detection (LOD) and limit of quantification (LOQ) were calculated using the formulas LOD = (3.3 × σ)/m and LOQ = (10 × σ)/m, respectively.

### 2.9 Statistical analysis

Data were analyzed from three individual experiments and presented as the mean ± standard error of the mean. Differences between the means of the individual groups were analyzed by one-way analysis of variance using GraphPad Prism 9.3.1 software (San Diego, CA, USA) followed by Dunnett’s test. p < 0.05 denoted statistical significance.

## 3 Results

### 3.1 Growth of hairy roots and molecular analysis

The hairy roots of *C. sativa* were successfully induced from a petiole with high response efficiency (>50%). They emerged after 16 days of transformation and grew well with the healthy appearance of the hairy root organ (**Figure 2B**). After the hairy roots grew in cefotaxime-free and auxin-free B5 medium, the lateral branches were cut and placed on the surface of the solid medium. They continued growing without a sign of bacterial contamination. In addition, the hairy roots were observed for continuous and horizontal growth in the liquid medium. These indicated the success of hairy root regeneration. To further confirm the success of the transformation event, genomic DNA was extracted and used as a template for *rolB* amplification by PCR using specific primers. The result revealed that the expected product size of 246 bp was obtained compared to the negative control (non-transformed root, **Figure 2C**), confirming that the hairy roots were induced by *R. rhizogenes* infection.

**Figure 3:**
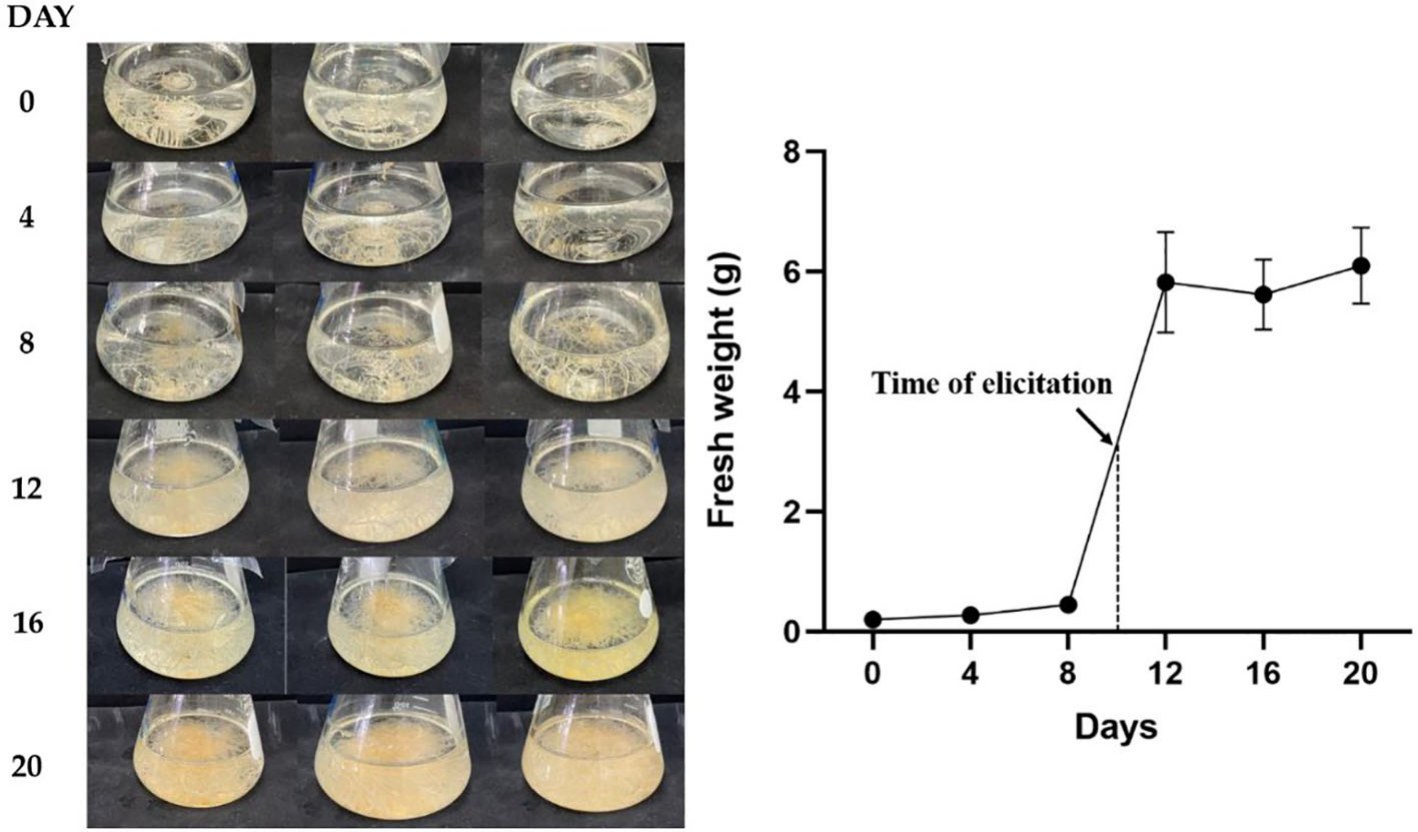
Analyses for the growth rate of transgenic hairy roots in B5 meduim for 20 days.

Hairy root growth was assessed on days 0, 4, 8, 12, 16, and 20 days (**Figure 3**). The fresh weight of the hairy root was determined until day 20 of subculture. The lag phase was observed between days 0 and 8, and the linear growth phase was observed on days 8–12. The highest growth was observed on day 12 and was followed by a stationary phase between days 12 and 20.

### 3.2 Chemical profile of the *in-vivo* grown roots and hairy roots of *C. sativa*

**Figure 4:**
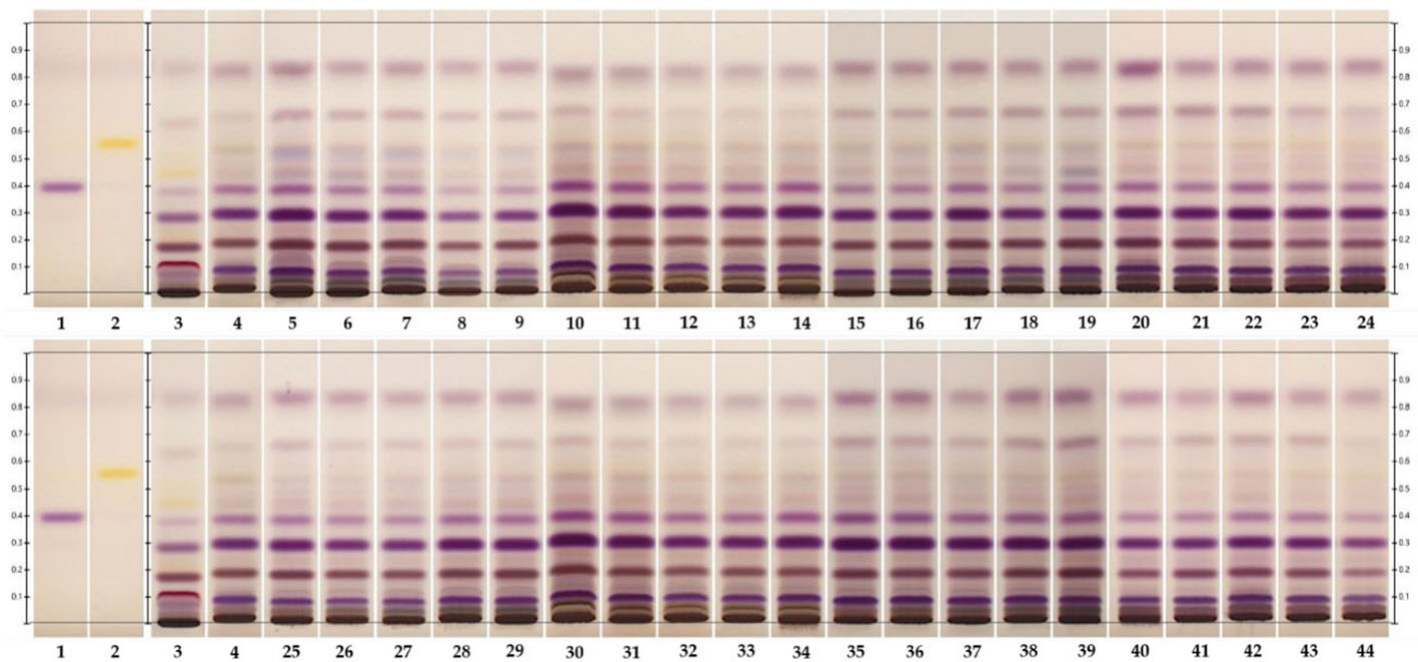
HPTLC fingerprints of *in- vivo* grown root and hairy root samples under white light after derivatization. Lane 1 and 2, epifriedelanol and friedelin reference standards, respectively; lane 3; hemp roots; lane 4; hairy roots; lane 5-9 and 25-29, hairy roots, supplemented with increasing concentrations of MeJA (0-100μM) for 3 and 6 days, respectively; lane 10-14 and 30-34; hairy roots supplemented with increasing concentrations of YE (0-100 mg/L) for 3 and 6 days, respectively; lane 15-19 and 35-39, hairy roots supplemented with increasing concentrations of SA (0-100μM) for 3 and 6 days; and lane 20-24 and 40-44, hairy roots supplemented with increasing concentrations of CHI (0-100μM) for 3 and 6 days, respectively.

**Figure 5:**
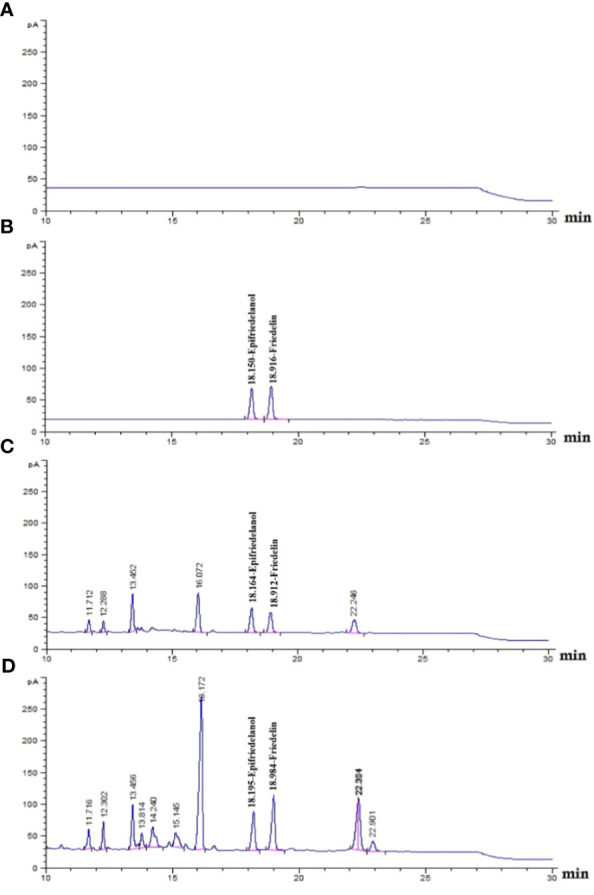
Chemical profiling if *in- vivo* grown roots and untreated hairy root of *C. sativa* roots using GC-FID. **(A)** chloroform run; **(B)** epifriedelanol and friedelin reference standards (t_r_ of 18.1 and 18.9 min, respectively); **(C)**, *in- vivo* grown roots hemp roots; **(D)**, hemp untreated hairy roots.

The detection of the triterpenoids friedelin and epifriedelanol was performed using HPTLC. The results showed that the reference standards for epifriedelanol and friedelin were detected as purple and yellow bands under white light after derivatization at the Rf values of 0.40 and 0.56, respectively (**Figure 4**, Lane 1–2). Similar patterns were observed for *in-vivo* grown roots and hairy root samples (**Figure 4**, Lane 3–4). However, the *in-vivo* grown roots root extract of *C. sativa* showed an additional dark purple band at Rf 0.12 and a weak purple band at Rf 0.54. The latter was not visible as the yellow band of friedelin, but this zone was detected in all hairy root extracts. Epifriedelanol was clearly detected in all extracts featuring a purple zone corresponding to the standard reference position at Rf 0.40 under white light after derivatization (**Figure 4**, Lane 3–44). Although the presence of epifriedelanol was obviously detected, the existence of friedelin was indistinct. The yellow band for the reference friedelin in all extracts displayed weak intensity. To ensure that the elicitors’ solvents did not affect the change of metabolites after the elicitation of hairy roots, the chemical profiles of hairy root samples treated with the elicitors’ solvents were observed. They were found to be the same as the control untreated hairy root cultures (data not shown). In addition, the target metabolites (friedelin and epifridelanol) were not detected in the medium analyzed by HPTLC, indicating that they were not released out of the roots (data not shown). As a result, the liquid medium was discarded. Furthermore, its chemical profile was analyzed by GC-FID to confirm the presence of both triterpenoids using chloroform as a baseline (**Figure 5A**). The chromatograms of the epifriedelanol and friedelin standards indicated retention times (t_r_) of 18.1 and 18.9 min, respectively (**Figure 5B**). The chemical profiles of *in-vivo* grown roots and hairy roots as determined by GC-FID featured similar peaks. Friedelin and epifriedelanol were produced in both natural and hairy root samples (**Figures 5C, D**). The hairy root extract featured more peaks (t_r_ between 14–15 min and 22.9 min) and also showed an interesting peak at t_r_ 16.1 min with high intensity.

### 3.3 The accumulation of triterpenoids in native and hairy roots

**Figure 6:**
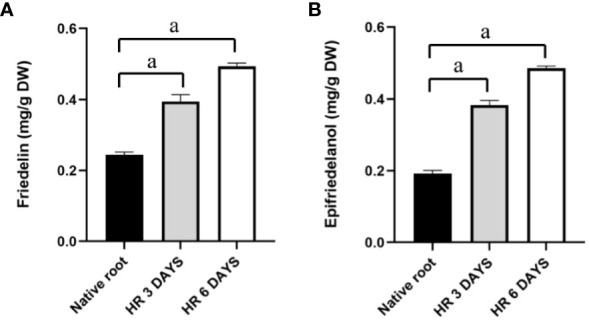
Friedelin and epifriedelanol content in hairy roots at 3 and 6 days. **(A)** friedelin content; **(B)** epifriedelanol content. “a” indicates statistical significance at p < 0.05 in comparison to native roots (n = 3).

The triterpenoid content in native roots was compared to that in untreated hairy roots on days 3 and 6. The result indicated that the friedelin levels in hairy roots on days 3 and 6 were 1.61- and 2.02-fold (0.394 and 0.494 mg/g DW, respectively) higher than those in native roots (0.245 mg/g DW), respectively (**Figure 6A**). Similarly, hairy roots on days 3 and 6 produced 2.0- and 2.54-fold more epifriedelanol, respectively (0.383 and 0.486 mg/g DW respectively) than native roots (0.192 mg/g DW, **Figure 6B**). In addition, hairy roots accumulated significantly higher amount of both triterpenoids day 6 than on day 3.

### 3.4 Effect of elicitors on triterpenoid accumulation

**Figure 7:**
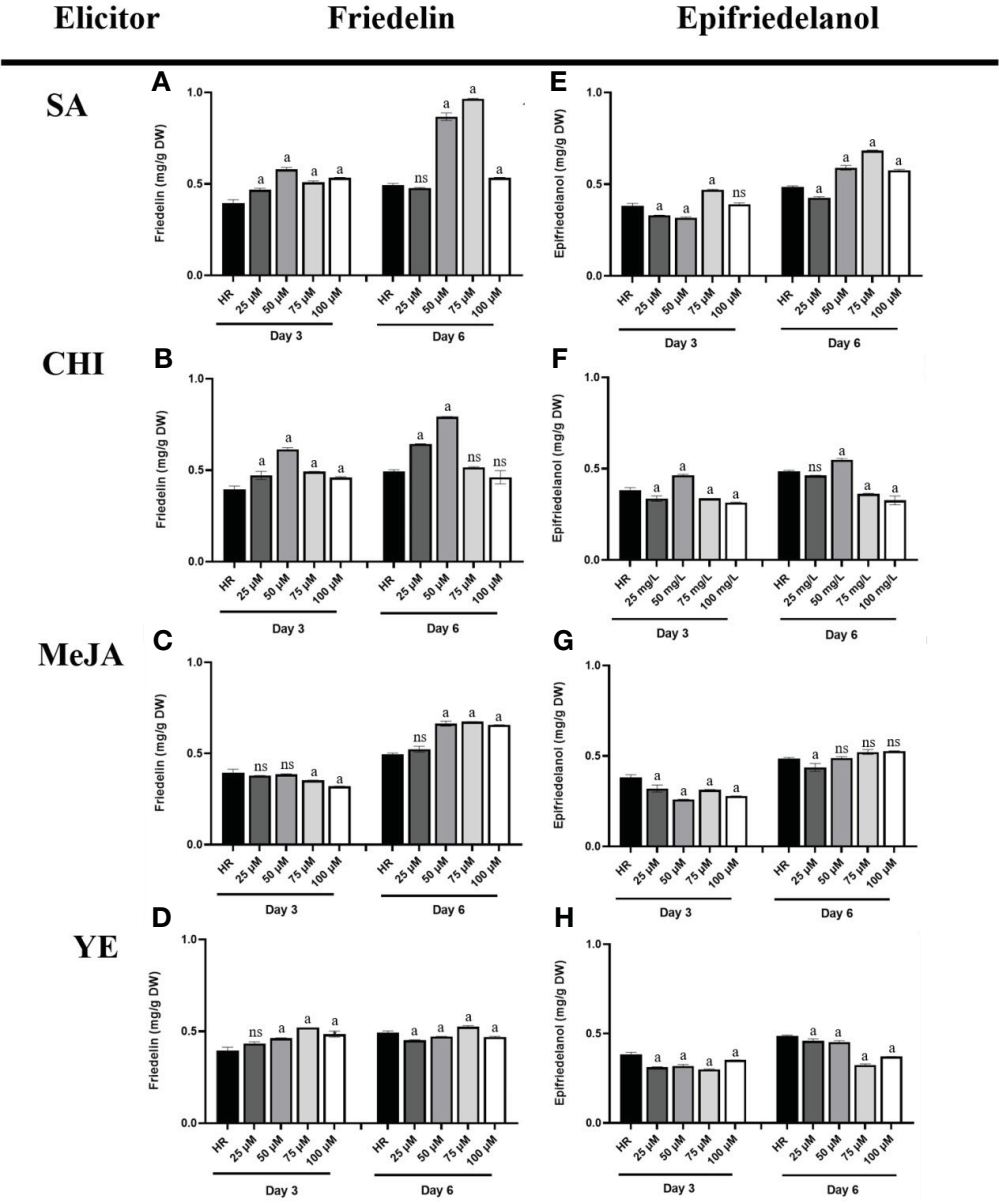
Friedelin and epifriedelanol content in hairy roots treated with different concentrations of various elicitors. Friedelin content in hairy roots elicited by SA **(A)**, CHI **(B)**, MeJA **(C)**, and YE **(D)**. Epifriedelanol content in hairy roots elicited by SA **(E)**, CHI **(F)**, MeJA **(G)**, and YE **(H)**. “a” indicates statistical significance at p < 0.05 in comparison to untreated hairy roots (HR), ns mean no significance (n = 3).

The effects of four elicitors (MeJA, SA, YE, and CHI) at various concentrations on triterpenoid production were investigated by GC-FID using untreated hairy roots as a control. The analytical method was validated following the ICH guidelines. In this study, the limit of detection (LOD) and limit of quantification (LOQ) for friedelin were 12.34 and 41.15 μg/mL, respectively while epifriedelanol showed LOD and LOQ at 42.82 and 42.73 μg/mL, respectively. The % recovery of friedelin and epifriedelanol was tested and showed at range of 95.66 - 103.26 and 96.10 - 107.95, respectively. The result was in the acceptable range and confirmed the reliability of the method. The elicitors were applied to the culture, and culture was continued for 6 days before harvest. To determine the effect of the elicitors on triterpenoid production, hairy roots were harvested on days 3 and 6 and subjected to analysis. The result showed that the friedelin content of hairy root cultures elicited with SA, CHI, and MeJA was higher on day 6 than on day 3 (**Figures 7A–C**), whereas friedelin content did not differ in hairy root cultures elicited with YE on days 3 and 6 (0.43–0.52 mg/g DW, **Figure 7D**). In this study, we found that among the four elicitors, SA (75 μM, day 6) was the most effective elicitor for friedelin production (**Figure 7A**), followed by CHI (50 µM, day 6, **Figure 7B**) and MeJA (75 µM, day 6, **Figure 7C**). SA at 75 μM for 6 days induced a 1.95-fold higher friedelin level than the control. Furthermore, CHI (50 μM) and MeJA (75 μM) on day 6 of elicitation increased friedelin production by 1.60 and 1.37-fold, respectively, versus the control (**Figures 7B, C**). The friedelin content was significantly lower in hairy root elicited with 100 μM SA and CHI on day 6, whereas it’s content on day 3 did not decrease significantly (**Figures 7A, B**). The friedelin content in hairy roots elicited with 50–100 μM MeJA remained at 0.65–0.67 mg/g DW, whereas YE treatment did not significantly alter friedelin levels (0.43–0.52 mg/g DW). Similarly, treated hairy root cultures featured a higher amount of epifriedelanol on day 6 than on day 3 (**Figures 7E–H**). Likewise, hairy roots treated with 75 μM SA on day 6 displayed the greatest increase in epifriedelanol production (1.4-fold versus the control, **Figure 7E**), followed by 50 µM CHI (1.1-fold, **Figure 7F**). This study found that friedelin and epifriedelanol levels ranged 0.377–0.967 and 0.302–0.685 µg/g DW, respectively, after 6 days of elicitation.

## 4 Discussion

In previous studies, hairy roots of *C. sativa* were induced in solid B5 medium supplemented with 4 mg/L naphthaleneacetic acid, and cannabinoid accumulation was observed (Farag and Kayser, 2015). Moreover, hairy roots of cannabis were developed to determine the choline and atropine accumulation (Wahby et al., 2006). However, triterpenoid content was overlooked. In this study, a comparative analysis of the content of triterpenoids (friedelin and epifriedelanol) in roots derived from natural sources and in elicitor-treated hairy roots was performed. Our present work represents the first report on the enhancement of triterpenoid accumulation in hairy root cultures of *C. sativa*.

In prior research, the friedelin was the primary triterpenoid in the roots of *C. sativa*, and its content ranged 0.83–1.35 (Jin et al., 2020) and 0.100–0.709 mg/g (Kornpointner et al., 2021) in those studies, in line with the present findings. However, the content of friedelin was much higher in untreated hairy roots than in native roots on days 3 and 6. In prior studies, the epifriedelanol content in *C. sativa* was lower than that of friedelin, ranging 0.33–0.92 (Jin et al., 2020) and 0.05–0.205 mg/g (Kornpointner et al., 2021), similar to the present results. Meanwhile, the epifriedelanol content was lower in native roots than in untreated hairy roots on days 3 and 6. However, the content of bioactive compounds probably depends on the location (Slatkin et al., 1971; Jin et al., 2020; Kornpointner et al., 2021).

No prior study examined the effects of abiotic and biotic elicitors on triterpenoid production in *C. sativa*. In this study, the accumulation of triterpenoids was assessed after 3 and 6 days of exposure to various concentrations of elicitors, namely MeJA, SA, CHI, and YE. The results showed that the triterpenoid content in elicited hairy roots was higher on day 6 than on day 3. SA was the most effective elicitor for both triterpenoids, followed by CHI. Both SA and CHI are important signal molecules in plant cells, and they are often used to induce the production of secondary metabolites in plants (Alcalde et al., 2022). SA and CHI have been found to increase the production of secondary metabolites in the hairy roots of several plants. For example, SA enhanced the content of withanolide A (48-fold), withanolide B (29-fold), withaferin A (20-fold), and withanone (37-fold) in root cultures of *Withania somnifera* (L.) Dunal (Sivanandhan et al., 2012). It has also been reported to increase the accumulation of terpenes by 8-fold in the hairy roots of *Andrographis paniculata* compared to that in control hairy roots (Sharmila and Subburathinam, 2013). In addition, 50 mg/L CHI increased the production of the triterpenoid cardenolide by 2.7-fold in the hairy roots of *Calotropis gigantea* (Sun et al., 2012), whereas 200 mg/L CHI increased saponin content by 4.55-fold in the hairy roots of *Psammosilene tunicoides* (Qiu et al., 2021).

Another elicitor tested in this study was MeJA, which has been reported to be the most effective inducer in many plants (Giri and Zaheer, 2016; Nabi et al., 2021). However, the compound only induced friedelin production in this study, and its effects were weaker than those of SA and CHI. Lastly, friedelin and epifriedelanol levels in hairy roots were not changed by YE treatment. Conversely, YE was reported to increase terpene production in other plants. For example, it induced the highest terpene production (tanshinone I, tanshinone IIA, and cryptotanshinone) in the hairy root of *Salvia castanea* Diels f*. tomentosa* Stib (Li et al., 2016).

In *C. sativa*, friedelin and epifriedelanol levels are highest in the roots, followed by the leaves and stem bark (Jin et al., 2020). Previous studies reported the friedelin and epifriedelanol content in cannabis inflorescences, leaves, stem bark, and roots (chemovar I, chemovar II, and chemovar III strains) using ethyl acetate with sonication (Jin et al., 2020). They showed that the leaves contained 0.05% of the total triterpenoid content, whereas triterpenoids were not detected in the inflorescences. Friedelin levels ranged 0.83–0.135 mg/g in roots and 0.33–1.0 mg/g in stem bark, whereas epifriedelanol levels ranged 0.33–0.92 mg/g in roots and 0.13–0.41 mg/g in stem bark. Another study revealed that cannabis root extracts (Futura 75 [France], Felina 32 [France], and Uso 31 [Netherlands]) obtained using supercritical CO_2_ contain friedelin and epifriedelanol content in the range of 0.100–0.709 and 0.05–0.205 mg/g, respectively (Kornpointner et al., 2021). These results differed from those in the present study. The extraction methods, solvents, plant, and location could affect the overall content of the compounds.

HPTLC fingerprint was developed for the preliminary screening of both triterpenoids, whereas GC-FID confirmed the presence of triterpenoids and determined their levels in the roots. Interestingly, hairy roots produced greater triterpenoid levels than natural roots. Upon the application of elicitors, hairy root cultures accumulated even higher quantities of triterpenoids. This study demonstrated that treated and untreated *C. sativa* hairy roots can be used in place of natural roots produce both triterpenoids because they produce higher triterpenoid levels and they grow rapid in medium that is not contaminated by heavy metals and pesticides. Therefore, this strategy would easily meet the standard requirements of herbal medicine.

## Data availability statement

The original contributions presented in the study are included in the article/supplementary material. Further inquiries can be directed to the corresponding author.

## Author contributions

KK: performing the experiments and writing - original draft. DR: Writing–review & editing. AB, TS, and PU: helped in the experiments. WD-E: Supervision and Writing–review & editing. SV: Conceptualization, Project administration, Supervision, Funding acquisition, Writing–review & editing. All authors contributed to the article and approved the submitted version.
